# Outcome of Cervical Lymph Nodes Dissection for Thyroid Cancer with Nodal Metastases: A Southeast Asian 3-Year Experience

**DOI:** 10.1155/2019/6109643

**Published:** 2019-02-28

**Authors:** Raymond Z. M. Lim, Juin Y. Ooi, Jih H. Tan, Henry C. L. Tan, Seniyah M. Sikin

**Affiliations:** ^1^Sultanah Aminah Hospital, Johor Bahru 80100, Malaysia; ^2^Hospital Raja Permaisuri Bainun, Ipoh 30450, Malaysia; ^3^Sultan Ismail Hospital, Johor Bahru 81100, Malaysia

## Abstract

**Introduction:**

Therapeutic nodal dissection is still the mainstay of treatment for patients with lymph node metastases in many centres. The local data, however, on the outcome of therapeutic LND remains limited. Hence, this study aims to inform practice by presenting the outcomes of LND for thyroid cancer patients and our experience in a tertiary referral centre.

**Methods:**

This is a single-centre retrospective observational study in a Malaysian tertiary endocrine surgery referral centre. Patients who underwent total thyroidectomy with lymph node dissection between years 2013 and 2015 were included and electronic medical records over a 3-year follow-up period were reviewed. The outcomes of different lymph node dissection (LND), including central neck dissection, lateral neck dissection, or both, were compared.

**Results:**

Of the 43 subjects included, 28 (65.1%) had Stage IV cancer. Among the 43 subjects included, 8 underwent central LND, and 15 had lateral LND while the remaining 20 had dissection of both lateral and central lymph nodes. Locoregional recurrence was found in 16 (37.2%) of our subjects included, with no statistical difference between the central (2/8), lateral (7/15), and both (7/20). Postoperative hypocalcaemia occurred in 7 (16.3%) patients, and vocal cord palsy occurred in 5 (11.6%), whereas 9 patients (20.9%) required reoperation. Death occurred in 4 of our patients.

**Conclusion:**

High recurrence and reoperative rates were observed in our centre. While the routine prophylactic LND remains controversial, high risk patients may be considered for prophylactic LND. The long-term risk and benefit of prophylactic LND with individualised patient selection in the local setting deserve further studies.

## 1. Introduction

Thyroid cancer is a common clinical problem which accounts for 3.1% of all new cancers, with a male-to-female ratio of 1:3 [1]. The global incidence of thyroid cancer also increased from 4.8 to 15.0 per 100,000 person-years in the period from 1975 to 2014. According to Othman et al., thyroid cancer accounts for 4.9% of the total cancers in Malaysia over a period of 11 years from 1994 to 2004 [2]. Lymph node metastasis among patients with thyroid cancer is commonly seen, especially with papillary thyroid cancer. The involvement of lymph nodes in thyroid cancer is associated with poorer outcomes including higher recurrence rates, reoperation rates, and poorer 5-year survival [3]. Surgical intervention is aimed at removing the thyroid cancer and its lymph node metastases, improving survival and patients' outcome.

Current American Thyroid Association (ATA) guidelines recommend the use of total thyroidectomy with the addition of therapeutic lymph node dissection (LND) in the presence of nodal involvement [4]. However, the long-term benefits of routine prophylactic cervical lymph node dissection remain unclear and are associated with an increase in morbidity including hypoparathyroidism and nerve injury. To the best of the author's knowledge, the local data on the outcome of LND remains limited. Hence, this study aims to inform practice by presenting the outcomes of LND for thyroid cancer patients and our experience in a tertiary referral centre.

## 2. Materials and Methods

A retrospective observational study was performed on all thyroid cancer patients who were admitted to Hospital Sultan Ismail, Johor Bahru, from 1^st^ January 2013 to 31^st^ December 2015. We included all subjects with thyroid cancer who underwent total thyroidectomy (TT) with LND. These data were obtained from the electronic records of Hospital Sultan Ismail, Johor Bahru, Malaysia (HSIJB)—a tertiary referral centre for endocrine malignancies for the southern region of Peninsular Malaysia. We obtained approval from the Medical Research and Ethics Committee (MREC), Ministry of Health Malaysia (MOH), and the National Medical Research Register (NMRR).

Details on subjects' demographics, diagnosis, operation performed, and the eventual outcomes were recorded into our electronic case record forms. The follow-up period for all subjects is three years postoperatively. Subjects included underwent TT with LND, including central nodes dissection, lateral nodes dissection, or both. Central neck dissections refer to the removal of the prelaryngeal, pretracheal, and paratracheal (Level VI) cervical lymph nodes whereas lateral neck dissection removes lymph nodes from level II through to level V [5]. Patients who did not undergo TT were excluded from this study. Staging included in this study was based on postoperative staging and histopathological examination after dissection with reference to the American Joint Committee for Cancer (AJCC) Cancer Staging Manual, seventh edition [6].

Statistical analysis of the variables was performed using IBM© SPSS© version 23. Discrete variables were expressed as percentage and continuous variables were expressed as means ± standard deviation (SD) or median with interquartile range (IQR) depending on normality. For frequency comparisons between types of LND and the outcomes, we used Chi-squared test and Fischer's Exact Test wherever applicable. A p-value of less than 0.05 is considered as statistically significant.

## 3. Results

A total of 43 subjects were included in this study, with the male to female ratio of 1:1.4. Mean age is 49.7 (SD 14.97). Among the subjects included, eight of them underwent central LND, 15 had lateral LND, and close to half (n= 20, 46.5%) had dissection of both central and lateral nodes. The demographic characteristics of the patients who underwent different lymph node dissections in this study including gender, age, cervical metastases, and the postoperative treatment are tabulated in Table 1. Notably, almost two-thirds (n=28; 65.1%) of our subjects had Stage IV cancer at the time of presentation.

Subjects' medical records were traced for three years with regular 6-month clinical examination, serum thyroglobulin monitoring, and radioiodine uptake testing to detect nodal or locoregional recurrence. Recurrence refers to the increase in serum Thyroglobulin level or new radioiodine uptake in the region of the thyroid bed and its surrounding cervical regions during the postoperative period [4].

Recurrence of thyroid cancer occurred in 16 (37.2%) patients (Table 2). Those with a higher staging (Stage IVa, IVb, IVc) did show a significant recurrence rate in this study, with 13 out of 28 Stage IV patients (46.4%) showing locoregional recurrence. On the other hand, different histological types did not show any significant difference in this study. When categorised based on the type of lymph node dissection, 2/8 (25%) of the central LND group, 7/15 (46.7%) in the lateral LND group, and 7/20 (35%) among those with both central and lateral LND had locoregional recurrence. No statistical significance of recurrence was observed among the patients who underwent different types of lymph node dissection.

When the papillary thyroid cancer group were investigated, there were no significant differences in its recurrence rate when tested against the various factors (Table 3).

**Figure 1:**
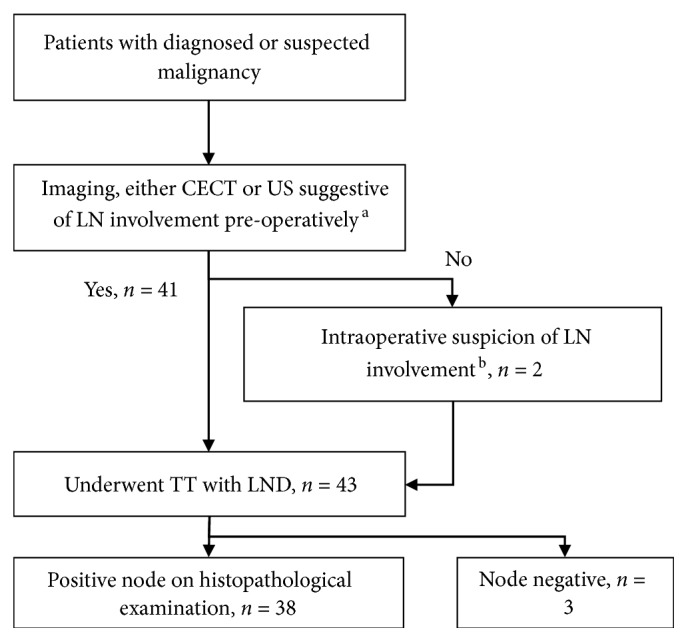
Yield of lymph node metastases among those who underwent total thyroidectomy (TT) and lymph node dissection (LND) based on imaging or intraoperative suspicion. CECT, contrast enhanced computed tomography; US, ultrasound; LN, lymph node; TT, total thyroidectomy; LND, lymph node dissection. ^a^Suspicious imaging findings include enlarged cervical lymph nodes with short axis more than 5 mm, calcifications, cystic changes, peripheral vascularity, rounded shape, hyperechogenicity, or obliteration of fatty hilum [6]. ^b^Suspicious intraoperative findings of cervical lymph node metastases include hard and ill-defined nodes, as well as adherence to surrounding tissue.

Our centre performs therapeutic lymph node dissection based on clinical suspicion for those who were node positive clinically based on examination, imaging, or intraoperative findings (Figure 1). Out of 43 patients who underwent LND, 41 showed cervical lymph node involvements on either ultrasonography or contrast-enhanced computed tomography (CECT). As per European Thyroid Association Guidelines, ultrasound findings suspicious of metastases include any enlarged cervical lymph nodes with short axis more than 5 mm, calcifications, cystic changes, peripheral vascularity, rounded shape, hyperechogenicity, or obliteration of fatty hilum [7] and warrant a dissection at our centre. The remaining two patients who did not show any lymph node sonographic changes were found to have suspicious lymph nodes intraoperatively and thus have their lymph nodes dissected as well (Figure 1). Among those who underwent lymph node dissection, 38 out of 43 (88.4%) were found to have actual metastases to the lymph nodes dissected. Suspicious intraoperative findings of cervical lymph node metastases include hard and ill-defined nodes, as well as adherence to surrounding tissue.

Postoperative hypocalcaemia occurred in 7 (16.3%) patients (Table 4). Fifty percent of patients underwent CND (n=4) compared to 15% (n=3) in patients with dissections of both central and lateral cervical lymph nodes. Other complications including hoarseness of voice, vocal cord palsy, and reoperation for recurrence for each group of patients are detailed in Table 4.

Death occurred in four of our patients; among them there were two papillary thyroid cancer patients, one follicular thyroid cancer patient, and one medullary thyroid cancer patient. One papillary thyroid cancer patient died of sepsis postoperatively. The other papillary thyroid cancer patient who died had extensive disease with tumour infiltrating the pretracheal region as well as brachiocephalic trunk and right subclavian artery. This results in difficult tumour dissection and despite undergoing TT, lateral LND, and postoperative chemotherapy and radiotherapy, the patient succumbed to death. The follicular thyroid cancer patient who died had an extensive metastasis preoperatively to the lungs and spine, and dissections were extremely difficult in view of extensive cervical lymph node metastases with infiltration to the right recurrent laryngeal nerve and surrounding structures found intraoperatively. Still another patient with medullary thyroid cancer died due to pulmonary embolism.

## 4. Discussion

The practice and outcome of different therapeutic neck dissections on thyroid cancer with cervical lymph nodes metastases in the current centre were presented. Therapeutic neck dissection is practised in our centre where only patients with either suspected or confirmed lymph node metastases undergo cervical lymph node dissection.

In our study, we found that the recurrence rate of 37.2% is relatively high (Table 2) compared to rates described in other studies. The rate of locoregional recurrence after a therapeutic neck dissection reported in other studies ranges between 28% and 38% [8, 9]. This could be explained by the subject selection, as the interest of this study focuses on lymph node dissection and it only included patients who underwent therapeutic lymph node dissection. Consequently, there is considerable proportion of patients presenting at later stages in our study (65.1%). The presence of nodal metastases prior to operation may be an attributive factor, where its relation has been described by others [10]. Factors such as complexity of surgery and extensive disease prior to surgery could contribute to higher recurrence, reoperative rates, and death in 2 out of 4 patients in our retrospective study. Previous local experience in Malaysia's tertiary referral centre did not reveal the incidence of different staging but showed that there is a median of 12-month duration of symptoms before first presentation to seek treatment [2]. The delay in treatment seeking may have contributed to the progress of disease and thus poorer outcome among local patients.

Our results showed no significant difference in terms of locoregional recurrence between the different types of neck dissection (central LND, 25.0%, lateral LND, 46.7%, and total cervical LND, 35.0%). In a retrospective study by Davidson [11] and his colleagues who examined 106 TT with therapeutic neck dissections, 40 patients (38%) suffered recurrence. Among those subjects included in their study, no significant difference was observed in Central LND group (26%), compared to that of Lateral LND (44%) and the combined central and lateral LND group (21%). Albeit no statistical significance observed, lateral LND group appeared to have higher recurrence compared to that of the other two groups. Out of the 43 patients in our study, 16 patients (37.2%) had locoregional recurrence.

Transient hypocalcaemia is commonly associated with central compartment dissection, attributed by the proximity of the inferior parathyroid gland and the location of Level VI cervical lymph nodes. This explains that the 16.3% of patients who experienced transient hypoparathyroidism were among the CND and combined group but not when LND was performed alone. Giordano et al. [12] have reported a significantly increased risk of transient hypoparathyroidism in patients who underwent a bilateral central LND (transient, 51.9%; permanent, 16.2%) as compared to those who only underwent TT (transient, 27.7%; permanent, 6.3%). In another retrospective review by Schuff et al., 12 out of 79 patients (16%) treated with therapeutic nodal dissection experienced transient hypoparathyroidism [13]. Roh et al. [14] in a 2007 study examined the outcomes of 82 patients who underwent neck dissection for thyroid cancer. Among the 82 patients, 9 patients (11%) were confirmed to have vocal cord palsy. In comparison, 5 out of 43 patients (11.6%) of our patients suffered new vocal cord palsy confirmed postoperatively.

Among the factors investigated, lateral nodes involvement prior to the initial surgery requiring lateral dissection of cervical nodes appeared to be associated with higher locoregional recurrence, albeit not statistically significant likely due to the small sample size. Therapeutic node dissection should be performed for patients with both biopsy-proven metastatic central and lateral cervical lymphadenopathy, but prophylactic cervical node dissection is only advised for clinically uninvolved central neck lymph nodes in patients with advanced primary tumours [4]. However, among those who underwent prophylactic lymph node dissection, lymph node metastases were found in as high as 39% of patients with clinically node-negative papillary thyroid cancer [15], suggesting the poor clinical sensitivity in detecting lymph node metastases. Hence, dissection based solely on clinical detection using examination and sonography may result in inaccuracy in staging. Clinical detection may, thus, result in false negative, delaying timely management and consequently result in higher locoregional recurrence.

While routine central LND performed with TT is associated with higher rate of postoperative temporary hypocalcaemia when compared to those who underwent TT alone and there was no difference between recurrent laryngeal nerve injury and locoregional recurrence rate [16], Gyorki et al. advised that the current available evidence is insufficient to conclusively recommend prophylactic central neck LND as a routine procedure [17]. Recent study, however, suggests that, in experienced hands, central LND can be performed with low morbidity, while improving node clearance in papillary thyroid cancer patients with lateral neck dissection [18]. Other authors, however, assert that the significantly higher rate of complications does not justify the routine use of prophylactic node dissection [19].

While this study presents an experience of the management of thyroid cancer patients with lymph node metastases, the small study limits its representability of the general experience of Malaysian hospitals in general. A multicentre prospective study with a longer follow-up period is warranted to further explore the findings identified in our study. This may provide more representative data of the experience in treating thyroid cancer with lymph node metastases in Malaysia.

## 5. Conclusion

This study presents a single centre experience on the practice among thyroid cancer patients with nodal metastases in a tertiary referral centre. Current study demonstrates a considerable locoregional recurrence rate in our three-year follow-up period. While the routine prophylactic LND remains controversial, high risk patients may be considered for prophylactic LND [4]. The long-term risk and benefit of prophylactic LND with individualized patient selection in the local setting deserve further studies.

## Figures and Tables

**Table 1:** Patients' demographic characteristics.

Characteristic		Type of Lymph Node Dissection, *N* (%)		
		CND, n=8	LND, n=15	BOTH, n=20
Age, mean (SD), in years		44.75 (16.4)	52.53 (12.4)	49.7 (16.3)
Gender				
	Male, *n* = 18 (41.9)	3 (16.7)	10 (55.6)	5 (27.8)
	Female, *n*= 25 (58.1)	5 (20.0)	5 (20.0)	15 (60.0)
TNM Staging^a^				
	I, *n*=12 (27.9)	4 (33.3)	2 (16.7)	6 (50.0)
	II, *n*=2 (4.7)	0 (0.0)	1 (50.0)	1 (50.0)
	III, *n*=1 (2.3)	1 (100.0)	0 (0.0)	0 (0.0)
	IVa, *n*=21 (48.8)	2 (9.5)	9 (42.9)	10 (47.6)
	IVb, *n*=4 (9.3)	0 (0.0)	3 (75.0)	1 (25.0)
	IVc, *n*=3 (7.0)	1 (33.3)	0 (0.0)	2 (66.7)
Extra Cervical Metastases upon presentation		2 (33.3)	1 (16.7)	3 (50.0)
RAI Ablation		6 (20.0)	11 (36.7)	13 (43.3)
Radiotherapy		0 (0.0)	4 (50.0)	4 (50.0)
Chemotherapy		0 (0.0)	2 (50.0)	2 (50.0)

Abbreviations: RAI Ablation, Radioactive ^131^Iodine Ablation; CND, central neck dissection; LND, lateral neck dissection; BOTH, both central and lateral neck dissection. ^a^TNM staging is based on American Joint Committee for Cancer (AJCC) Cancer Staging Manual Seventh Edition [6].

**Table 2:** Subgroup analysis of those who had recurrence in the 3-year follow-up period.

N = 43		Recurrence n (%)		OR (95% CI)	*p*
		Yes, n=16 (37.2)	No, n= 27 (62.8)		
Types of LND					
	Central LND n=8	2 (25.0)	6 (75.0)		0.569
	Lateral LND n=15	7 (46.7)	8 (53.3)	-	
	Central + Lateral LND n=20	7 (35.0)	13 (65.0)		
Histological types					
	Papillary thyroid cancer n=30	11 (36.7)	19 (63.3)		
	Follicular thyroid cancer n=7	2 (28.6)	5 (71.4)	-	0.755^a^
	Anaplastic thyroid cancer n=1	1 (100.0)	0 (0.0)		
	Medullary thyroid cancer n=5	2 (40.0)	3 (60.0)		
TNM Staging^b^					
	I, n=12	2 (16.7)	10 (83.3)		
	II, n=2	1 (50.0)	1 (50.0)		
	III, n=1	0 (0.0)	1 (100.0)	-	0.047^a^
	IVa, n=21	8 (38.1)	13 (61.9)		
	IVb, n=4	4 (100.0)	0 (0.00)		
	IVc, n=3	1 (33.3)	2 (66.7)		
Tumour margin clear	Yes n=29	10 (34.5)	19 (65.5)	0.71 (0.19-2.59)	0.594
	No n=14	6 (42.9)	8 (57.1)		
LN margin clearance	Yes n = 28	11 (39.3)	17 (60.7)	1.29 (0.35-4.81)	0.700
	No n=15	5 (33.3)	10 (66.7)		
Radioiodine usage	Yes, n =30	13 (43.3)	17 (56.7)	2.55 (0.58-1.12)	0.307^a^
	No, n =13	3 (23.1)	10 (76.9)		
Radiotherapy	Yes, n = 8	4 (50.0)	4 (50.0)	1.92 (0.41-9.01)	0.443^a^
	No, n = 35	12 (34.3)	23 (65.7)		
Chemotherapy	Yes, n = 4	2 (50.0)	2 (50.0)	1.79 (0.23-14.08)	0.621^a^
	No, n = 39	14 (35.9)	25 (64.1)		

LND, lymph node dissection; LN, lymph node; ^a^P value calculated using Fisher Exact Test. ^b^TNM staging is based on American Joint Committee for Cancer (AJCC) Cancer Staging Manual Seventh Edition [6].

**Table 3:** Subgroup analysis of those who had recurrence in the 3-year follow-up period among papillary thyroid cancer group (n=30).

N = 30		Recurrence *n* (%)		OR	*p*
		Yes, n=16 (37.2)	No, n= 27 (62.8)	(95% CI)	
Types of LND					
	Central LND n= 6	1 (16.7)	5 (83.3)		0.320^a^
	Lateral LND n= 9	5 (55.6)	4 (44.4)	-	
	Central + Lateral LND n= 15	5 (33.3)	10 (66.7)		
Tumour margin clear	Yes n= 19	6 (31.6)	13 (68.4)	0.55 (0.12-2.56)	0.696^a^
	No n= 11	5 (45.5)	6 (54.5)		
LN margin clearance	Yes n= 19	7 (36.8)	12 (63.2)	1.02 (0.22-4.76)	1.000^a^
	No n= 11	4 (36.4)	7 (63.6)		
Radioiodine usage	Yes, n= 26	11 (42.3)	15 (57.7)	-	0.268^a^
	No, n= 4	0 (0.00)	4 (100.0)		
Radiotherapy	Yes, n= 3	2 (66.7)	1 (33.3)	4.00 (0.32-50.0)	0.537^a^
	No, n= 27	9 (33.3)	18 (66.7)		
Chemotherapy	Yes, n= 2	2 (100.0)	0 (0.00)	-	0.126^a^
	No, n= 28	9 (32.1)	19 (67.9)		

LND, lymph node dissection; ^a^P value calculated using Fisher Exact Test.

**Table 4:** Complications among patients that underwent lymph node dissection.

*N* = 43		Types of LND, *n* (%)		
		Central, *n*=8	Lateral, *n*=15	Dissection of both central and lateral nodes, *n*=20
Hypocalcaemia	Yes, n=7 (16.3)	4 (50.0)	0 (0.0)	3 (15.0)
	No, n=36 (83.7)	4 (50.0)	15 (100.0)	17 (85.0)
Vocal cord palsy	Yes, n=5 (11.6)	1 (12.5)	2 (13.3)	2 (10.0)
	No, n=38 (88.4)	7 (87.5)	13 (86.7)	18 (90.0)
Re-operative rate	Yes, n=9 (20.9)	2 (25.0)	4 (26.7)	3 (15.0)
	No, n=34 (79.1)	6 (75.0)	11 (73.3)	17 (85.0)
Death	Yes, n=4 (9.3)	0 (0.0)	2 (13.3)	2 (10.0)
	No, n=39 (90.7)	8 (100.0)	13 (86.7)	18 (90.0)

LND, lymph node dissection.

## Data Availability

The research data used to support the findings of this study have not been made available because the authors do not have permission from the Ministry of Health Malaysia to share them.
